# Tuberculosis in Sheltered Homeless Population of Rome: An Integrated Model of Recruitment for Risk Management

**DOI:** 10.1100/2012/396302

**Published:** 2012-02-01

**Authors:** Patrizia Laurenti, Stefania Bruno, Gianluigi Quaranta, Giuseppe La Torre, Antonio G. Cairo, Pierangela Nardella, Giovanni Delogu, Giovanni Fadda, Tommaso Pirronti, Salvatore Geraci, Salvatore Pelargonio, Francesco N. Lauria, Delia Goletti, Gualtiero Ricciardi

**Affiliations:** ^1^Department of Hygiene, Università Cattolica del Sacro Cuore, Largo Francesco Vito, 00168 Rome, Italy; ^2^Department of Public Health and Infectious Diseases, Sapienza University of Rome, Piazzale Aldo Moro, 00185 Rome, Italy; ^3^Department of Microbiology, Università Cattolica del Sacro Cuore, Largo Francesco Vito, 00168 Rome, Italy; ^4^Department of Radiologic Sciences, Università Cattolica del Sacro Cuore, Largo Francesco Vito, 00168 Rome, Italy; ^5^Caritas Health Service, Via Marsala, 00185 Rome, Italy; ^6^Acisel Day Centre, Via Verzuolo, 00166 Rome, Italy; ^7^National Institute of Infectious Diseases, Lazzaro Spallanzani, Via Portuense, 00149 Rome, Italy

## Abstract

The authors show the results of an integrated model for risk management of tuberculosis in a sample of sheltered homeless in Rome. Tuberculin skin test (TST) was used for evaluating the prevalence of latent infection (LTBI). In TST positives, expectorate was collected and chest X-ray was achieved. Multiple logistic regression analysis was performed to investigate determinants of infection. Out of 288 recruited subjects, 259 returned for the TST reading; 45.56% were positive and referred to a specialized center; 70 accessed the health facility and completed the clinical pathway. The risk factors associated to LTBI were male gender (OR = 3.72), age over 60 years (OR = 3.59), immigrant status (OR = 3.73), and obesity (OR = 2.19). This approach, based on an integrated social network, guarantees high adherence to screening (89.93%), allowing patients testing positive for latent tuberculosis infection to be diagnosed and rapidly referred to a specialized center.

## 1. Introduction

Together with other social factors, homelessness is one of the greatest risk factors for the acquisition of latent tuberculosis infection (LTBI) and active Tuberculosis (TB) [1]. In recent years, a few surveys have been carried out in Europe to evaluate the LTBI and TB prevalence in homeless people: the existing data (Rotterdam 29%, London 0.5%) show that adequate screenings and treatment pathways are needed to reduce the spread of *Mycobacterium tuberculosis* in overcrowded shelters and in the community [2, 3].

In Italy, the incidence is extremely low in the general population. In 2008, crude incidence rate was 3.8/100,000 for those born in Italy and 50–60/100,000 for those born abroad. In recent years, the incidence of pulmonary tuberculosis seems stable and around 5-6 cases per 100,000 residents. However, the concentration of the majority of TB cases was observed among certain risk groups: alcohol abusers and drug users, homeless people, HIV-infected people, and young migrants [4–6].

**Figure 1:**
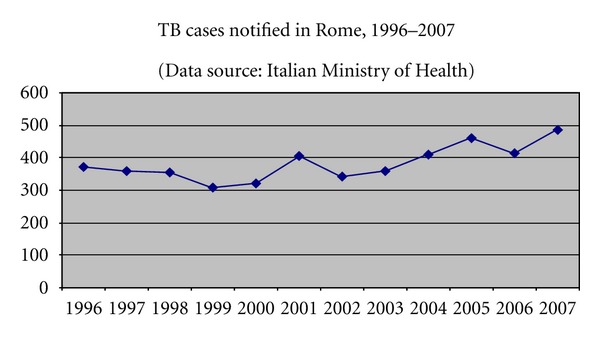
Linear diagram showing TB cases notified in Rome in the time-period 1996–2007 (source of the data: [7]).

In Rome, in the time period 1996–2007, an annual average of 382.5 TB cases were notified (11.55% of the total notified in Italy). In spite of a decreasing trend from 1996 to 1999, in the years 2006 and 2007, the trend is again increasing, with an annual average of 448.5 cases, corresponding to a rate of about 16.6/100.00 population (Figure 1) [7].

A considerable increase of people living on the road has been reported in recent years: in 1998, a figure of 5,000 homeless people was estimated in Rome, but nowadays the expected number is about 23,000 [8], and the numbers alone could probably be a contributing factor explaining the increased rate of TB cases reported in the period 2002–2005 (409 cases in 2004 and 460 cases in 2005, up from 341 in 2002). 

Epidemiological studies of homeless populations have reported the prevalence rate of 1.2%–6.8% for active TB [9, 10]. No data about the incidence of TB in homeless people are to date available in Rome. 

Better understanding of the characteristics of homeless people with TB disease is important for creating strategies to reduce TB incidence in this high-risk population [11].

Based on these assumptions, we enrolled homeless people from two shelters in Rome. A cross-sectional study was carried on with the following aims:

1. to assess acceptance to came back for TST reading in a sample of homeless people recruited from two shelters in Rome (subjects who returned for Tuberculin Skin Test (TST) reading, after 72 hours),
2. to measure the prevalence of skin-positive results, assumed as LTBI,
3. to investigate the associations between social risk factors and TST positive results,
4. to evaluate the access to a public specialized outpatient clinic for initiating an early therapeutic pathway.

## 2. Methods

The protocols of this research were cleared by the local ethical committee.

### 2.1. Population and Setting

The eligible individuals—male and female, Italians and foreigners, adults ≥ 18 years old—were recruited from two homeless refuges in Rome (named Caritas and Acisel) in the time period November 2006-November 2007.

These two shelters were selected by convenience among all the others in Rome. Individuals were recruited according to HUD (US Department of Housing and Urban Development) homeless definition, as “an individual who lacks a fixed, regular,…, and adequate night-time residence…” [12].

The sample size was calculated using 2004 available data, the most recent ones existing when the protocol of the study was drafted, taking into account the homeless number in Rome, fixing the confidence level at 95%, estimating the LTBI prevalence as 25%, and considering a 90% study compliance. According to these parameters, 276 individuals were eligible to be recruited.

A detailed anamnesis was collected using a form to record data about markers and risk factors: gender, age, height, and weight (for body mass index (BMI)), origin, past TB diagnosis and therapy, immigration condition (both irregular an regular condition, without and with National health assistance, resp.), smoking habits, alcohol abuse (according to *Diagnostic and Statistical Manual of Mental Disorders (DSM-IV)*) and drugs use.

Previous *Bacillus Calmette Guerin* (BCG) vaccination was investigated, with a specific question and an inspection verifying the presence of any scar on the deltoid muscle.

### 2.2. TST and Other Laboratory Tests

The TST (Biocine test PPD 5 UI) was performed by medical physicians in the surgery at the refuges, after informing the subjects about the study and obtaining their understanding and written consent by a translated form.

TST was read after 72 hours. According to the American Thoracic Society standards, which have provided new recommendations for targeted tuberculin testing and treatment regimens for persons with latent tuberculosis infections (LTBI), a reaction with an induration diameter >10 mm and with normal chest X-ray was considered as positive and we classified this as LTBI to send the subject towards a clinical pathway, managed by a specialized hospital [13].

Only positive subjects were invited to undertake a chest X-ray and to provide three sputum samples on alternate days. The chest X-ray was performed with an MPX+ (GE Healthcare, Italy) portable equipment for radiography and read by two different radiologists. The sputum was collected with a safe disposable device (Sputum Collection System, Becton Dickinson, USA).

The bacterioscopic examination (Ziehl-Nielsen staining) and cultures in solid (Lowenstein-Jensen) and liquid media (an automatic system MGIT, Becton Dickinson, USA), were performed by the Microbiology Unit, according to internal QC procedures.

Moreover, PCR was prepared to identify and confirm *M. tuberculosis *(Probetec, Becton Dickinson, USA).

In the case of *M. tuberculosis *isolates, a phenotypic study of drug resistance to streptomycin, isoniazid, ethambutol, and rifampicin and the genetic typing with random amplification of primers designed technique (RAPD), based on PCR technique, was prepared.

The positive subjects to TST were referred to INMI (National Institute for Infectious Diseases “Lazzaro Spallanzani”) in Rome for clinical evaluation.

### 2.3. Statistical Analysis

Univariate analysis by logistic regression was performed using the chisquare test and the Mann-Whitney test for assessing differences between groups for categorical and quantitative variables, respectively.

A multivariate analysis was performed using the dichotomous variable *LTBI risk* as the dependent variable and the following variables as independent variables: gender (female as reference group), marital status (single as reference group), nationality (foreigners as reference group), nutritional status (no obesity as reference group), and age category (age under 60 as reference group). The results are presented as odds ratios (OR) and 95% confidence intervals (95% CI).

A stepwise approach (backward elimination procedure) was followed, using the method suggested by Hosmer and Lemeshow. The variables were excluded from the model until a *P*  value > 0.10. The goodness of fit of the model was assessed using the Hosmer and Lemeshow test. 

The statistical significance was set at *P* ≤ 0.05.

The statistical analysis was conducted using SPSS software (release 12.0).

## 3. Results

From the two homeless shelters, 288 subjects were recruited; for each subject, a data collection form was filled in and a TST performed. In the same period, 133 expectorates were collected and 46 chest X-rays performed. Among all recruited subjects, 259 came back for the TST reading, with an acceptance rate of 89.93%. Of these, 204 subjects (78.8%) were males and 55 (21.2%) females; 31 (12%) were over 60 years old. As far as marital status, 122 subjects (47.1%) were single, 68 (26.2%) married, 51 (19.7%) divorced or separated, and 16 (6.2%) widows/widowers (Table 1). 

Regarding the social features, 171 (66%) were immigrants and 65 (25.1%) were employed during the recruitment period. Among them, 108 (41.7%) declared to be registered within the Italian National Health Service, while 17 (2.7%) reported to be foreigners temporarily present. Regarding educational level, 13 (5%) had no education title, 124 (47.9%) had completed Primary school, 102 (39.4%) had completed Secondary school, and 12 (4.6%) were graduates.

Four subjects (1.5%) reported a previous TB diagnosis. Regarding the smoking habit, 147 (56.8%) were smokers, 99 (38.2%) nonsmokers, and 13 (5%) former smokers.

Twenty-two individuals (8.5%) declared to be alcohol abusers and 10 (3.9%) exalcohol abusers. Most of the participants declared not to be illicit drug users, though three subjects (1.1%) declared to be drug-users and 8 (3.1%) exdrug users.

Out of the 259 compliant subjects, 141 (54.44%) were TST negative and 118 (45.56%) were positive, therefore suffering from LTBI. Among these, 18 (15.4%) were over 60 years old, 16 (13.6%) were females and 102 (86.4%) were males.

No chest X-ray, sputum, and culture sample was positive, respectively, for TB and Mycobacterium tuberculosis.

In Table 2, the results of TST and the native land are reported: most people were Italians (33.2%) and Romanians (32%), as regards the TST positives, 27.1% were Italians and 39.8% Romanians.

All TST-positive subjects were referred to a specialized outpatient clinic and 70 of them (59,32%) accessed the health facility and completed the diagnostic pathway to receive the appropriate therapy.

The other 48 were lost to the study because of their homelessness condition, as all homeless were not stably residential in the shelter.

One subject (3.86‰) showed clinical suspicion of active disease and was promptly referred to hospital.

The results for LTBI risk, according to the multiple logistic regression analysis performed in order to discover the relationship with the risk factors, are shown in Table 3. Immigrant status, male gender, older age (over 60 years), and obesity were significant risk factors for developing LTBI, as shown from the OR and CI values.

## 4. Discussion and Conclusions

In large European metropolitan areas, for example, in London [14], in Rotterdam [4], in Marseilles [15], and in the Warminsko-Mazurskie Province of Poland [16], a high prevalence of Tb was still observed in homeless people.

Immigration, under-nutrition [17], smoking [18], diabetes [19], and alcohol misuse [20] are individual risk factors that can double or triple the risk of active TB development and all these conditions are often associated with homelessness.

In these perspectives, the results of our study indicate a prevalence of LTBI of 45.56% among the homeless and of 3.86‰  for active TB.

The low rate of active TB, compared to cited experiences, could be interpreted as a sign of protection and full immune-competence of this group of subjects. It may be hypothesised that the incidence of active TB in this cohort of LTBI-positive homeless persons may be explained by the good quality of conditions available for the homeless living in Italian shelters, which warrant good nutritional status and, therefore, low risk of progression to active disease. Nevertheless, the remarkably high LTBI prevalence observed may indicate that the homeless may have a higher risk of contracting *M. tuberculosis* infection because of overcrowded environments or because they shelter in a community where the greater chance of an active TB patient transmitting the microorganism is not promptly recognized.

In this perspective, finally, immigrants should not be considered as spreaders of the disease but as persons at risk, and appropriate public health approaches should be oriented towards them, aimed at inclusion and at the promotion of barrier-free prevention and care measures [21]. 

In Italy, health is a constitutional right for everyone, migrants included, independent of their judicial condition. Nevertheless, in practice, a high level of inequality affects access to healthcare services, as this is noted when we compare the Italian population with migrants. Available data reveal a substantial failure of health and social policies for integration [22].

In 2008, European Centre for Disease Control (ECDC) published a framework action plan to fight TB in the European Union (EU), which recognizes the concentration of TB in “hard to find” and “hard to treat” populations as a major challenge to TB control efforts across the EU and encourages EU institutions to collaborate with partners to identify and disseminate good practice models for TB control [23].

On these foundations, our study applies this model of TB control in advance and for the first time in Rome and produced a high compliance amongst the homeless population. In fact, a high adherence to screening was observed (89.93%) if compared with a 65% adherence to TST reading registered in a preventive therapy programe carried out in an inner city population in Atlanta in 1994–1996 [24] and with a maximum adherence rate of 84% using economic incentive strategy to encourage test reading adherence in a high-risk population [25].

Our data confirm that active surveillance is an excellent tool for prevention. Active research in crowded areas gave the possibility to 59.32% of the positive subjects to access health care facilities and to complete the clinical pathway.

The strength of our experience was the opportunity to reach people in shelters (where people sleep and eat) and to start a clinical activity within the same place, with highly motivated staff, in order to facilitate access by the most disadvantaged persons.

Nevertheless, the compliance to the TST reading decreased from 89.93% to a 59.32% clinical compliance when health and social support was missing and people were invited to go to hospital by themselves. The active role of a social network also should be strengthened with the aims of prevention, diagnosis, treatment, and followup. For example, the Dutch TB control model is based on close collaboration between hospitals and municipal public health TB [23].

Moreover, guidelines should take into account the peculiarities of risk groups for whom health is not a priority [26].

**Study Limitations**

Firstly, concerning internal validity, a possible bias could have been introduced by a 10% of refusal to come back for the test reading.

In any case, persons who came back for TST reading are substantially similar to the ones who did not regarding two important variables: age (*P* = 0.639) and gender (*P* = 0.718). As such, we expect that subjects who did not return for a TST reading did not influence the statistical analysis.

Secondly, some aspects could also affect external validity. In fact, the homeless evaluated were recruited in two shelters in Rome and this aspect could represent an important issue for the generalization of this study's results to the whole Italy. Perhaps the study's findings may not be generalized to all homeless shelters in Rome because the socio-demographic and socio-economic characteristics of homeless people living in the other shelters are unknown.

A further limit of our study could be related to the impossibility to use the latest interferon gamma release assays (IGRAs) because of blood sampling difficulties in the studied population, that would have been useful in the diagnosis of recent infection.

Lastly, atypical infection and effects of the BCG used in the country may impact on the results of TST.

## Figures and Tables

**Table 1:** Characteristics of participants according to the TST result.

Variable	TST + *N* (%)	TST − *N* (%)	*P*
Gender			
Male	102 (50)	102 (50)	0.006
Female	16 (29.1)	39 (70.9)	
Age (years)			
<60	99 (43.8)	127 (56.2)	0.135
≥60	18 (58.1)	13 (41.9)	
Marital status			
Single	49 (40.2%)	73 (59.8%)	
Married	38 (55.9%)	30 (44.1%)	0.158
Divorced or separated	22 (43.1%)	29 (56.9%)	
Widowers	9 (56.2%)	7 (43.8%)	
Immigrant			
Yes	85 (49.7%)	86 (50.3%)	0.062
No	33 (37.5%)	55 (62.5%)	
Italian National Health Service			
No	59 (46.5%)	68 (53.5%)	
Yes	47 (43.5%)	61 (56.5%)	0.831
Unknown	1 (33.3%)	2 (66.7%)	
Temporary Present Foreigners			
No	31 (49.2)	32 (50.8)	
Yes	7 (41.2)	10 (58.8)	0.473
Not Known	3 (75)	1 (25)	
Educational level			
No instruction title	5 (38.5)	8 (61.5)	
Primary school	54 (43.5)	70 (56.5)	0.585
Secondary school	51 (50)	51 (50)	
Degree	5 (33.3)	7 (66.7)	
Employment			
No	86 (44.6)	107 (55.4)	0.661
Yes	31 (47.7)	34 (52.3)	
Previous TBC diagnosis			
No	114 (46.2)	133 (53.8)	0.115
Yes	3 (75)	1 (25)	
Smoking habit			
No smoker	42 (42.4)	57 (57.6)	
Smoker	72 (49)	75 (51)	0.328
Former smoker	4 (30.8)	9 (69.2)	
Alcohol abuse			
No	98 (44.3)	123 (55.7)	
Yes	11 (52.4)	10 (47.6)	0.743
Exalcohol abuser	5 (50)	5 (50)	
Illicit drug use			
No	105 (45.5)	126 (54.5)	
Yes	1 (33.3)	2 (66.7)	0.833
Former drug user	3 (37.5)	5 (62.5)	
Obesity			
No	87 (42.9)	116 (57.1)	0.096
Yes	30 (55.6)	24 (44.4)	

**Table 2:** Results of the TST by the native land.

Native Land	No. of subjects	TST + *N* (%)	TST − *N* (%)
Italy	86	32 (37.2)	54 (62.8)
Romania	83	47 (56.6)	36 (43.4)
Afghanistan	22	9 (40.9)	13 (59.1)
Eritrea	14	6 (42.9)	8 (57.1)
Others	54	24 (44.4)	30 (55.6)

Total	259	118 (45.6)	141 (54.4)

**Table 3:** Results of the multivariate approach performed in order to find statistically significant determinants of LTBI. (OR: Odds Ratio; CI: Confidence Interval).

	Univariate analysis	Multivariate analysis
	OR (95% CI)	OR (95% CI)
Native land		
African (referent)	1	
Eastern Mediterranean	0.68 (0.22–2.16)	
Europe	1.42 (0.51–3.94)	
America or Asia or Western Pacific	0.27 (0.04–1.90)	
Gender		
Female	1	1
Male	5.06 (2.29–11.17)	3.72 (1.83–7.58)
Age group		
<60 years	1	1
≥60 years	3.64 (1.40–9.51)	3.59 (1.40–9.21)
Immigrant status		
No	1	1
Yes	5.13 (2.15–12.25)	3.73 (1.89–7.39)
Employment		
No	1	
Yes	1.00 (0.52–1.94)	
Obesity		
No	1	1
Yes	1.91 (0.93–3.91)	2.19 (1.10–4.35)
Education		
Lower (primary school)	1	
High (secondary school or degree)	0.84 (0.52–1.35)	
Marital status		
Single	1	
Married	1.81 (0.91–3.61)	
Divorced, separated, or widowers	1.63 (0.77–3.43)	
